# Physiological Actions of Fibroblast Growth Factor-23

**DOI:** 10.3389/fendo.2018.00267

**Published:** 2018-05-28

**Authors:** Reinhold G. Erben

**Affiliations:** Department of Biomedical Sciences, University of Veterinary Medicine Vienna, Vienna, Austria

**Keywords:** fibroblast growth factor-23, Klotho, vitamin D, 1α-hydroxylase, bone mineralization, phosphate metabolism, alkaline phosphatase

## Abstract

Fibroblast growth factor-23 (FGF23) is a bone-derived hormone suppressing phosphate reabsorption and vitamin D hormone synthesis in the kidney. At physiological concentrations of the hormone, the endocrine actions of FGF23 in the kidney are αKlotho-dependent, because high-affinity binding of FGF23 to FGF receptors requires the presence of the co-receptor αKlotho on target cells. It is well established that excessive concentrations of intact FGF23 in the blood lead to phosphate wasting in patients with normal kidney function. Based on the importance of diseases associated with gain of FGF23 function such as phosphate-wasting diseases and chronic kidney disease, a large body of literature has focused on the pathophysiological consequences of FGF23 excess. Less emphasis has been put on the role of FGF23 in normal physiology. Nevertheless, during recent years, lessons we have learned from loss-of-function models have shown that besides the paramount physiological roles of FGF23 in the control of 1α-hydroxylase expression and of apical membrane expression of sodium-phosphate co-transporters in proximal renal tubules, FGF23 also is an important stimulator of calcium and sodium reabsorption in distal renal tubules. In addition, there is an emerging role of FGF23 as an auto-/paracrine regulator of alkaline phosphatase expression and mineralization in bone. In contrast to the renal actions of FGF23, the FGF23-mediated suppression of alkaline phosphatase in bone is αKlotho-independent. Moreover, FGF23 may be a physiological suppressor of differentiation of hematopoietic stem cells into the erythroid lineage in the bone microenvironment. At present, there is little evidence for a physiological role of FGF23 in organs other than kidney and bone. The purpose of this mini-review is to highlight the current knowledge about the complex physiological functions of FGF23.

## Introduction

In the year 2000, gain-of-function mutations in fibroblast growth factor-23 (FGF23) were identified as the genetic cause of autosomal dominant hypophosphatemic rickets (ADHR), an inherited renal phosphate-wasting disease (1). In the following years, FGF23 turned out to be the long-sought “phosphatonin” that had already been postulated in the 1980s, when parabiosis experiments in hypophosphatemic *Hyp* mice had shown that the renal phosphate wasting and the hypophosphatemia in these mice were caused by a factor circulating in the blood (2).

Fibroblast growth factor-23 is a 32 kDa glycoprotein mainly produced in bone by osteoblasts and osteocytes under physiological circumstances. FGF23 is inactivated by cleavage at the ^176^RXXR^179^ site, a site that is mutated in ADHR patients. Together with FGF19 and FGF21, FGF23 belongs to the group of endocrine FGFs (3). All endocrine FGFs require the co-receptors α- and β-Klotho for high-affinity binding to the ubiquitously expressed FGF receptors (FGFR1-4) in target cells (4–7). The co-receptor needed for binding of FGF23 to FGF receptors is transmembrane or soluble αKlotho (4, 8). Among the four different FGFRs, FGF receptor-1c (FGFR1c) is probably the most important FGFR for FGF23 signaling, at least under physiological conditions (4, 9). αKlotho enhances the binding affinity of FGFR1c to FGF23 by a factor of approximately 20 (6). The “c” in FGFR1 stands for a splice variant which occurs in FGFR1, 2, and 3.

The principal action of FGF23 on mineral metabolism that led to its discovery as a hormone is the suppressive effect on phosphate reabsorption from the urine (10, 11). In addition, FGF23 suppresses the synthesis of the vitamin D hormone, 1α,25-dihydroxyvitamin D_3_ [1,25(OH)_2_D_3_], in the kidney (10, 11). It is now well known that diseases characterized by excessive blood concentrations of intact FGF23 lead to renal phosphate wasting and inappropriately low-circulating 1,25(OH)_2_D_3_ levels in patients with a normal kidney function (12). Examples of human disorders associated with elevated intact FGF23 are ADHR with a defective cleavage site of FGF23, X-linked hypophosphatemic rickets (XLH), and autosomal recessive hypophosphatemic rickets 1 (ARHR1) caused by overproduction of FGF23 in bone, and tumor-induced osteomalacia caused by FGF23-producing tumors (12). The molecular mechanism underlying the increased bony FGF23 secretion in XLH and ARHR1 patients is still unclear. In terms of factors that may drive FGF23 secretion, the common denominator in both diseases is impaired bone mineralization. XLH is caused by loss-of-function mutations in *PHEX* (13). It has been shown in *Hyp* mice, the murine model of XLH, that lack of the endopeptidase PHEX leads to accumulation of osteopontin and ASARM (acidic serine- and aspartate-rich MEPE-associated motif) peptides in the matrix, which both inhibit mineralization (14–17). ARHR1 is caused by loss-of-function mutations in dentin matrix protein-1, which is required for normal mineralization of bone (18). It is currently believed that the excessive osteocytic and osteoblastic FGF23 secretion in both diseases is either driven by the impaired mineralization of the extracellular matrix, which may be detected by matrix-embedded bone cells through a putative sensing mechanism that may involve FGF receptors (19, 20), or by an altered set point for phosphate sensing in bone cells (21, 22). Circulating intact FGF23 is also elevated in patients with chronic kidney disease (CKD), and can reach blood levels as high as 1,000-fold above the normal range (23, 24). Although elevated intact FGF23 may help to maintain normophosphatemia in early stages of CKD, serum phosphate levels typically increase at later stages of the disease despite very high-serum intact FGF23. Therefore, in the setting of impaired kidney function, the phosphaturic action of FGF23 is not able to correct the hyperphosphatemia in more advanced CKD.

Collectively, there is very good evidence that gain of FGF23 function results in renal phosphate wasting in patients with normal kidney function. However, what is the role of FGF23 in normal physiology? The purpose of this mini-review is to answer this question, and to highlight the current knowledge about the complex physiological functions of FGF23 in mice and men.

## Physiological Functions of FGF23 in the Kidney

Knockout experiments in mice have revealed that the paramount physiological function of FGF23 is not its phosphaturic function, but its suppressive role in the control of renal 1α-hydroxylase (CYP27B1) transcription, the key enzyme for 1,25(OH)_2_D_3_ synthesis. Notably, in the absence of the ligand FGF23 or of its co-receptor αKlotho, the stringent endocrine control of 1α-hydroxylase transcription fails, leading to inappropriately high expression and activity of this enzyme. The sequel of 1α-hydroxylase overexpression are elevated 1,25(OH)_2_D_3_ levels, causing hypercalcemia, hyperphosphatemia, ectopic calcifications, impaired bone mineralization, and early lethality in α*Klotho* and *Fgf23* deficient mice (25–27). The major function of 1,25(OH)_2_D_3_ in mineral metabolism is the stimulation of intestinal calcium and phosphorus absorption. The crucial role of 1,25(OH)_2_D_3_ overproduction in mediating the phenotype of α*Klotho*^−/−^ and *Fgf23^−/−^* mice is underscored by the well documented finding that ablation of vitamin D signaling almost completely rescues the phenotype of *Fgf23^−/−^* and α*Klotho*^−/−^ mice (28–30). In analogy to the phenotype of α*Klotho* and *Fgf23* deficient mice, humans with loss-of-function mutations in *FGF23* or α*Klotho* are characterized by elevated circulating vitamin D hormone levels and soft tissue calcifications (31–34), corroborating the mouse data.

**Figure 1:**
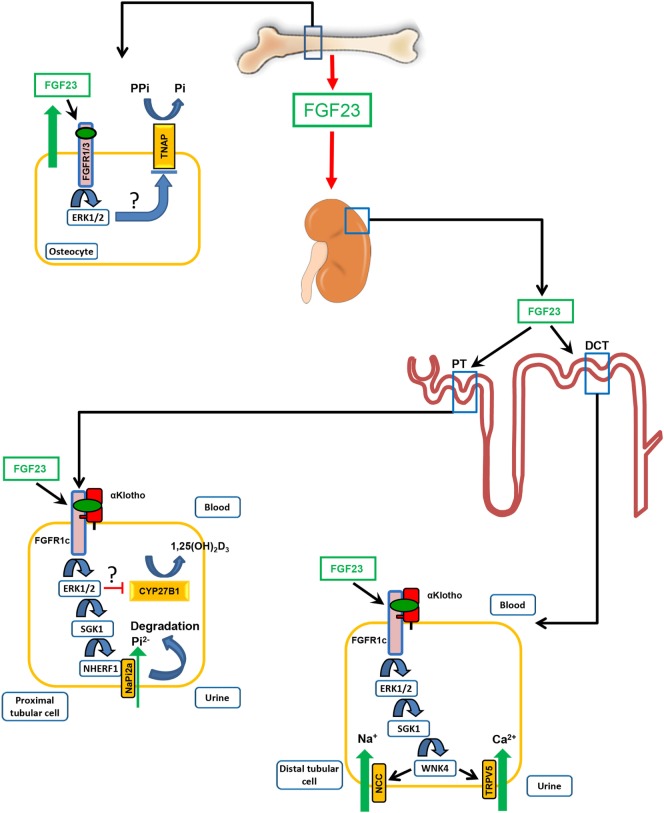
Physiological functions of fibroblast growth factor-23 (FGF23). FGF23 is mainly produced in bone cells, osteoblasts, and osteocytes. One of the main target organs of the hormone FGF23 is the kidney. In the kidney, FGF23 acts on proximal and distal convoluted renal tubules. Binding of blood-borne FGF23 to FGF receptor-1c (FGFR1c) requires the presence of the co-receptor αKlotho. In renal proximal tubules (PT), FGF23 inhibits phosphate (Pi) re-uptake and expression of 1α-hydroxylase (CYP27B1), the rate-limiting enzyme for vitamin D hormone (1α,25(OH)_2_D_3_) production. The FGF23-mediated suppression of 1α-hydroxylase transcription involves extracellular signal-regulated kinase-1 and 2 (ERK1/2) activation. However, the exact signaling pathway downstream of ERK1/2 is unknown. The inhibition of phosphate reabsorption in proximal renal tubules by FGF23 is mediated through activation of ERK1/2 and serum/glucocorticoid-regulated kinase-1 (SGK1), leading to phosphorylation of the scaffolding protein Na^+^/H^+^ exchange regulatory cofactor (NHERF)-1. NHERF-1 phosphorylation triggers internalization and degradation of the sodium-phosphate cotransporter NaPi-2a, so that less NaPi-2a is available in the apical membrane for phosphate uptake from urine. In distal convoluted tubules (DCT), FGF23 increases reabsorption of calcium and sodium by increasing the apical membrane abundance of the epithelial calcium channel transient receptor potential vanilloid-5 and of the sodium-chloride cotransporter NCC through a signaling cascade involving ERK1/2, SGK1, and with-no-lysine kinase-4 (WNK4). FGF23 locally produced by osteocytes is an auto-/paracrine inhibitor of bone mineralization by down-regulating tissue non-specific alkaline phosphatase (TNAP) transcription in a αKlotho-independent fashion *via* FGFR1- or FGFR3-mediated activation of ERK1/2. The signaling pathway downstream of ERK1/2, leading to suppression of TNAP transcription, is not known. TNAP is essential for normal mineralization of bone by cleaving the mineralization inhibitor pyrophosphate (PPi).

Despite the pivotal physiological importance of the FGF23-mediated suppression of 1α-hydroxylase transcription, the knowledge of the intracellular signaling pathway involved in this regulation is still fragmentary. Expression of 1α-hydroxylase is mainly localized in proximal renal tubules. Proximal and distal renal tubules express the co-receptor αKlotho as well as FGFR1, 3, and 4, but only little FGFR2 (35, 36). All FGFRs are receptor tyrosine kinases, initiating intracellular phosphorylation cascades after ligand-induced dimerization (37). Mice with a specific deletion of *Fgfr1* in proximal renal tubules are resistant to the FGF23-induced suppression of 1,25(OH)_2_D_3_ production (9). Therefore, FGFR1c is probably the predominant FGFR mediating the suppressive effects of FGF23 on renal tubular 1,25(OH)_2_D_3_ synthesis (Figure 1). However, to a lesser extent, FGFR3 and 4 may also be involved, because genetic ablation of *Fgfr3* and *Fgfr4* increases renal 1α-hydroxylase expression in *Hyp* mice, which are characterized by increased endogenous FGF23 production (38). There is good evidence that the FGF23-mediated suppression of 1α-hydroxylase transcription involves extracellular signal-regulated kinase-1 and -2 (ERK1/2) activation (39, 40), but the exact signaling pathway downstream of ERK1/2 is unknown (Figure 1). Interestingly, the FGF23-mediated control of the transcriptional activity of the 1α-hydroxylase gene occurs through regulatory elements located in introns of the neighboring *Mettl21b* gene (41). However, the transcription factor(s) involved in this regulation are currently unknown. It is only clear in this context that FGF23 regulates 1α-hydroxylase in a 1,25(OH)_2_D_3_ and vitamin D receptor (VDR) independent manner (42).

It is well established that parathyroid hormone (PTH) and FGF23 regulate 1α- and 24-hydroxylase (CYP24A1) expression reciprocally. FGF23 suppresses 1α-hydroxylase, but induces 24-hydroxylase expression. PTH has the opposite effects. 1α-hydroxylation represents metabolic conversion of the precursor 25-hydroxyvitamin D into the biologically active hormone, whereas 24-hydroxylation is an inactivation pathway (43). 1,25(OH)_2_D is known to be a strong inducer of 24-hydroxylase (43), stimulating its own degradation. Whether FGF23 is a direct regulator of 24-hydroxylase transcription has been a controversial issue for many years. Some reports in *Fgf23*^−/−^ mice (26) as well as in wild-type mice treated with recombinant FGF23 (11) suggested that FGF23 signaling may directly induce 24-hydroxylase, whereas experiments in VDR knockout mice suggested that the FGF23-mediated regulation of 24-hydroxylase is not direct, but depends on the VDR (42, 44). The latter notion has been confirmed by recent evidence showing that, in contrast to the 1,25(OH)_2_D_3_-mediated induction, both the FGF23-mediated induction and the PTH-mediated suppression of 24-hydroxylase are completely lost in 1α-hydroxylase knockout mice (41). This finding strongly suggests that the FGF23 and PTH-mediated regulation of 24-hydroxylase expression are entirely indirect through altered 1,25(OH)_2_D_3_ synthesis and subsequent changes in VDR-regulated promoter activity of 24-hydroxylase.

As mentioned above, FGF23 promotes renal phosphate excretion by inhibiting cellular phosphate re-uptake from the urine in proximal renal tubules (Figure 1). Through a signaling cascade involving the αKlotho/FGFR1c receptor complex, ERK1/2, and serum/glucocorticoid-regulated kinase-1 (SGK1), FGF23 signaling induces the phosphorylation of the scaffolding protein Na^+^/H^+^ exchange regulatory cofactor (NHERF)-1 which in turn leads to internalization and degradation of the sodium-phosphate cotransporters NaPi-2a and NaPi-2c (36, 45, 46). Notably, 4-week-old *Fgf23^−/−^*/VDR^Δ/Δ^ (*Fgf23*/VDR) and α*Klotho*^−/−^/VDR^Δ/Δ^ (*Klotho*/VDR) compound mutant mice lacking *Fgf23* or *Klotho* and a functioning VDR are not hyperphosphatemic (28, 29, 47). Hyperphosphatemia is only seen in older, more slowly or non-growing *Fgf23*/VDR compound mutant mice beyond 3 months of age (30, 47), suggesting that the phosphaturic effect of FGF23 is physiologically less essential compared with the 1α-hydroxylase-suppressing effect, at least in mice. Both the phosphaturic and the 1,25(OH)_2_D_3_-lowering effect of FGF23 protect against hyperphosphatemia: the first effect directly through increased elimination of phosphate, and the second effect indirectly through reduced intestinal phosphate absorption. In addition, because 1,25(OH)_2_D_3_ and phosphate stimulate FGF23 secretion in bone (12), the phosphaturic and 1,25(OH)_2_D_3_-lowering effects of FGF23 form a negative feedback loop between bone and kidney.

In recent years, it has become clear that FGF23 is not only a regulator of vitamin D and phosphate metabolism, but also directly influences calcium and sodium handling in the distal nephron in the kidney (Figure 1). Skeletally mature *Fgf23*/VDR and *Klotho*/VDR compound mutant mice are characterized by renal calcium wasting (48), as well as by renal sodium wasting and subsequent hyponatremia, hypovolemia, and hypotension (49). Similar to proximal renal tubules, the FGFR1c/Klotho complex appears to be the most important receptor complex in the distal nephron, because distal tubular-specific deletion of *Fgfr1* recapitulates the renal calcium wasting seen in *Fgf23*/VDR compound mutant mice (9). In distal tubular epithelium, FGF23 regulates the apical membrane abundance of the epithelial calcium channel transient receptor potential vanilloid-5 (TRPV5) and of the sodium-chloride cotransporter NCC through a signaling cascade involving ERK1/2, SGK1, and with-no-lysine kinase-4 (48, 49). *Fgf23* and *Klotho* deficient mice are characterized by a downregulation of distal tubular TRPV5 and NCC membrane expression, leading to renal calcium and sodium wasting, despite counter-regulatory increases in circulating PTH and aldosterone (48–50). These findings indicate that the calcium- and sodium-conserving functions of FGF23 in distal renal tubules are of physiological relevance. Indeed, the increased renal conservation of calcium may help to maintain blood calcium levels despite the suppression of 1,25(OH)_2_D_3_ synthesis induced by upregulated FGF23 secretion. In single *Fgf23* and *Klotho* knockout mice, the calcium-conserving function of FGF23 is masked by the profound upregulation of 1,25(OH)_2_D_3_ production and subsequent hypercalcemia.

Parathyroid hormone and FGF23 have partially overlapping functions in proximal and distal renal tubules. Both hormones inhibit phosphate reabsorption in proximal renal tubules by targeting NHERF-1 phosphorylation (36, 45, 46), and increase calcium reabsorption in distal renal tubules by targeting expression and/or open probability of TRPV5 (48, 51). Albeit the signaling mechanisms are different, the proximal and distal renal target molecules of PTH and FGF23 are the same. An interesting finding in this context is that absence of FGF23 signaling in *Fgf23* deficient mice causes partial renal resistance to the phosphaturic and calcium-conserving actions of PTH (50). *Vice versa*, a reduction in PTH signaling in human patients with hypoparathyroidism has been shown to induce partial resistance to the phosphaturic actions of FGF23 (52, 53). Therefore, both hormones interact, and an important physiological function of FGF23 may be to enable normal responsiveness to PTH signaling in the kidney and also in bone (50).

Taken together, lessons learned from knockout mouse models have revealed that the most important physiological function of FGF23 is not its phosphaturic effect but the downregulation of vitamin D hormone production. It is likely that the exquisite sensitivity of the homeostatic system regulating 1α-hydroxylase transcription to lack of FGF23 signaling is caused by the absence of other suppressive hormones which might be able to effectively counter-balance FGF23 deficiency. In *Fgf23* and *Klotho* deficient mice, the suppression of PTH secretion observed in these mice is insufficient to control transcription of renal 1α-hydroxylase. In contrast, lack of the phosphaturic, as well as of the calcium- and sodium-conserving functions of FGF23 can at least partially be compensated by the phosphaturic and calcium-conserving hormone PTH, and by the sodium-conserving hormone aldosterone. Therefore, the pivotal importance of FGF23 signaling for the control of 1α-hydroxylase transcription might be considered as a systems biology problem. This problem may also have implications for the treatment of patients with antibodies against FGF23 or with small molecules blocking the FGF23 signaling pathway. The therapeutic window for these treatments is relatively narrow, and requires close monitoring of calcium and phosphorus metabolism to avoid toxic side effects (54, 55).

## Physiological Functions of FGF23 in Bone

Fibroblast growth factor-23 may also have physiologically relevant functions in bone on bone mineralization and on hematopoiesis. We recently reported that FGF23 is a powerful suppressor of transcription of tissue non-specific alkaline phosphatase (TNAP) mRNA in bone cells in a Klotho-independent manner (56) (Figure 1). TNAP is essential for the regulation of bone mineralization by cleaving the mineralization inhibitor pyrophosphate which is secreted by osteoblasts to prevent premature mineralization of osteoid (57). Based on experiments with pharmacological FGFR inhibitors, we concluded that the FGF23-induced, Klotho-independent suppression of TNAP mRNA abundance in primary murine osteoblasts is mainly mediated through FGFR3 (56). In contrast, Shalhoub et al. (58) reported that FGF23 suppresses TNAP expression in mouse osteoblast-like cells in an FGFR1-dependent manner, and that this effect could be enhanced by soluble Klotho. Thus, it awaits further clarification whether the FGF23-mediated suppression of TNAP in bone cells is mainly mediated through FGFR1, FGFR3, or both. Klotho expression in bone is very low (33, 59). Therefore, it is unlikely that Klotho expression in bone cells is sufficient to enhance FGF23 binding to FGFRs in osteoblasts and osteocytes. However, it can be assumed that due to the local production of FGF23 in osteocytes, the concentration of FGF23 within the canalicular system is high enough for auto-/paracrine, Klotho-independent signaling through FGFRs in bone. Hence, locally produced FGF23 may not only contribute to impaired mineralization under the conditions of excessive bony FGF23 secretion such as in *Hyp* mice (60), but may also serve as a physiological inhibitor of bone mineralization by downregulating TNAP expression. In line with this notion, we found an upregulation of *Tnap* mRNA abundance in *Fgf23* deficient *Fgf23*/VDR compound mutant mice compared with wild-type and VDR control mice (56). However, the relevance of this mechanism in the context of physiological ranges of FGF23 secretion remains to be shown.

*Fgf23* deficient mice are characterized by increased erythropoiesis (61). Conversely, injection of recombinant FGF23 into normal mice suppresses erythropoiesis (61), and inhibition of FGF23 signaling alleviates the suppression of erythropoiesis in mice with excessive FGF23 blood levels due to renal failure (62). Therefore, FGF23 may be a physiological regulator of erythroid lineage commitment in the bone microenvironment. However, the signaling mechanisms underlying this effect are currently not known, and further studies are needed to demonstrate the relevance of this effect in relation to the physiological regulation of erythropoiesis by the renal hormone erythropoietin.

## Physiological Functions of FGF23 in Other Organs?

It is interesting to note that the first description of FGF23 was actually in thalamic nuclei in the murine brain (63). However, data about possible functions of FGF23 in the brain are still scarce. It was reported that high concentrations of FGF23 may interfere with neuronal ramification and may increase synaptic density in cultures of hippocampal neurons (64), but very little is known about potential physiological functions. *Fgf23*/VDR compound mutant mice do not have an overt CNS phenotype until older ages (30), but more elaborate behavioral or cognitive tests have never been done.

The parathyroid gland is one of the organs abundantly expressing αKlotho (33, 65), making it a potential target tissue for FGF23. However, conditional knockout mice with a parathyroid-specific deletion of αKlotho show normal circulating intact PTH levels (65). In addition, global *Fgf23*/VDR and α*Klotho*/VDR mutant mice at young ages do not show differences in PTH blood concentrations compared with VDR mutant mice (28, 29). Only at older ages, PTH secretion is upregulated in *Fgf23*/VDR and α*Klotho*/VDR mutant mice, relative to VDR controls, in response to chronic renal calcium wasting and partial PTH resistance (50). Hence, although high-FGF23 blood concentrations may suppress PTH secretion in a Klotho-independent fashion in rodents (65, 66), it is unlikely that FGF23 signaling has an important role in the physiological regulation of PTH secretion.

Although the heart may be an important target tissue at supraphysiological FGF23 concentrations, promoting cardiomyocyte hypertrophy in CKD patients by a Klotho-independent signaling pathway (67, 68), FGF23 is not expressed in the normal heart, and heart function is normal in *Fgf23*/VDR mutant mice (69). These findings suggest that FGF23 does not have a functional role in the heart under physiological circumstances.

Taken together, there is only little evidence that FGF23 has a role in normal physiology in organs other than kidney and bone.

## Conclusion

The purpose of this mini-review is to highlight the current knowledge about the physiological functions of the bone-derived hormone FGF23. Excessive circulating intact FGF23 levels result in renal phosphate wasting under the conditions of a normal kidney function. However, knockout mouse models have shown that the most important physiological function of FGF23 is not the phosphaturic effect, but the suppressive effect on renal 1α-hydroxylase expression. The absence of FGF23 or of its co-receptor αKlotho results in deregulated renal 1α-hydroxylase expression and vitamin D hormone production, which cannot be compensated by other endocrine systems. Moreover, FGF23 has several additional physiological functions, which include inhibition of renal phosphate reabsorption, increased conservation of calcium and sodium in the kidney, support of a normal responsiveness of the kidney to PTH, and regulation of bone mineralization. Although excessive FGF23 may target many non-canonical tissues, there is currently little evidence for a role of FGF23 in the normal physiology of organs other than kidney and bone.

## Author Contributions

The author confirms being the sole contributor of this work and approved it for publication.

## Conflict of Interest Statement

The author declares that the research was conducted in the absence of any commercial or financial relationships that could be construed as a potential conflict of interest.
